# Psychosocial Distress of Patients with Psoriasis: Protocol for an Assessment of Care Needs and the Development of a Supportive Intervention

**DOI:** 10.2196/resprot.8490

**Published:** 2018-02-07

**Authors:** Jördis Maria Zill, Jörg Dirmaier, Matthias Augustin, Sarah Dwinger, Eva Christalle, Martin Härter, Ulrich Mrowietz

**Affiliations:** ^1^ Department of Medical Psychology University Medical Center Hamburg-Eppendorf Hamburg Germany; ^2^ Institute of Health Care Research in Dermatology and Nursing University Medical Center Hamburg-Eppendorf Hamburg Germany; ^3^ Psoriasis Center at the Department of Dermatology University Medical Center Schleswig-Holstein, Campus Kiel Kiel Germany

**Keywords:** psoriasis, psychosocial distress, care needs, supportive intervention

## Abstract

**Background:**

Psoriasis is a chronic inflammatory disease that is often associated with a number of somatic and mental comorbidity. Patients with psoriasis show an increased risk of depression and (social) anxiety.

**Objective:**

The aims of this study are 1) to explore the psychosocial distress of patients with psoriasis and to assess their care needs; and 2) to develop a supportive intervention based on the prior results.

**Methods:**

A multi-stage design with four phases combining quantitative and qualitative methodology will be used and conducted in two centers. 1) A scoping review and focus groups will be used to design a questionnaire to assess the psychosocial distress and care needs of the patients. 2) The questionnaire developed in phase 1 will be used in a cross-sectional survey to assess the extent of psychosocial distress and supportive care needs in 400 patients with psoriasis. 3) A systematic review and meta-analysis will be conducted to identify psychosocial and psychoeducational interventions for patients with psoriasis and to describe their effectiveness. 4) Based on the results of the phases 2 and 3 a manualized supportive intervention will be developed and the feasibility and acceptance of the intervention will be assessed.

**Results:**

Currently, phase 1 of the project has been completed and the recruitment for phase 2 has been started. The systematic review and meta-analysis of phase 3 are conducted simultaneously to phase 2 and results are expected soon. Phase 4 has not been started yet.

**Conclusions:**

The expected results of this study will show the extent of psychosocial distress of patients with psoriasis in Germany and supplement previous research with findings about the supportive care needs of this patient group. Moreover, the developed intervention will help to address the psychosocial support needs of patients with psoriasis. Research shows that psychosocial support is strongly needed.

## Introduction

Psoriasis is one of the most common chronic inflammatory skin diseases with a prevalence of 1 to 3 % in western industrial countries. In Germany about 2 million patients are affected [1,2]. The most common type of psoriasis is “psoriasis vulgaris” also called “plaque psoriasis.” It is assumed that about 25% of patients have moderate to severe disease, and a relevant proportion needs a lifelong treatment [3]. Studies indicate that psoriasis often is associated with other medical conditions, especially in severe cases with a long history of the disease [4]. Known comorbidity includes psoriatic arthritis (PsA) [5]; inflammatory bowel disease [6]; cardiovascular disease [7]; and diabetes [8], influencing morbidity and mortality [9,10]. Increased rates of comorbidity are already found in young children and adolescents [11]. In addition to somatic comorbidity psoriasis can be associated with psychosocial stress and mental illness. Patients with psoriasis show an increased risk of depression, anxiety, and suicidal ideation [12-15]. Especially an early onset of psoriasis increases the risk for depression and social anxiety [14,16]. Moreover, alcohol consumption and nicotine abuse seems to be greater in patients with psoriasis than in the general population [17,18].

Reasons for the psychosocial burden of patients with psoriasis are experienced stigmatization in social situations, the workplace, difficulties with body image, self-esteem, and self-concept [14,19-22]. These factors can be substantial components leading to impaired health-related quality of life (HrQoL) [23]. Other predictors of HrQoL impairments are pruritus [24,25], the time needed for daily treatment and treatment dissatisfaction [26]. Accordingly, patients show a large variety of needs related to disease management which go far beyond symptomatic treatment and include a plurality of psychosocial aspects [27].

Research suggests that psychosocial stress is not just a consequence of psoriasis but can also be involved in the exacerbation of the symptoms [28]. Reich et al [29] found that the itch intensity experienced during psoriasis exacerbation correlated positively with stressful events one month earlier. The concept of the “brain-skin-axis” describes the interaction between mental aspects, immune system, and cutaneous inflammation. In patients with psoriasis, psychosocial stress can worsen the condition, which increases disease-associated and experienced stress, impairs quality of life, and increases psychosocial strain and comorbidity [28]. The individual stress reactivity can affect both adherence as well as treatment response [28]. Besides the individual stress reactivity, age, sex, psychosocial, disease-specific, and treatment-specific factors predict the adherence or compliance [30]. Also in a study by Eskin et al, psoriasis patients showed lower scores in social problem-solving skills as well as higher degrees in negative problem orientation and impulsive-careless problem-solving style compared to healthy controls [31], which indicates that those patients might profit from interventions like problem-solving trainings.

For these reasons, current concepts of psoriasis management include screening for mental comorbidity and recommend interdisciplinary teamwork and psychosocial support if applicable [4].

Until now, different reviews subsumed studies on the effects of psychosocial interventions on psoriasis patients. Larsen et al [32] reviewed nine randomized controlled trials (RCTs), quasi-randomized trials, and controlled clinical trials of patient education and self-management interventions on disease severity and HrQoL and found that little evidence is available. They point out that compared with other chronic conditions, only few effective disease-specific tailored educational programs for psoriasis are available [32]. A narrative review found an overall positive effectiveness of existing psychological and/or educational interventions in psoriasis especially in psychological and HrQoL outcomes [33]. However, they stated that the strength of evidence to support the effectiveness of the published interventions was limited due to methodological weakness in the included studies and suggest further research and RCTs to increase validity of intervention studies. Despite a clear need and some existing evidence-based psychosocial treatments, access to psychological and psychosocial interventions within dermatological services remains limited [34]. Moreover, since evidence on psychosocial interventions is still equivocal, it can be concluded that it is important to find out more about the individual information and support needs of these patients to design interventions that are especially tailored to patient preferences. Therefore, the aims of the described study are:

1. To assess the psychosocial distress of patients with psoriasis
2. To investigate their needs for information and psychosocial support
3. To develop a psychosocial intervention to educate and support psoriasis patients
4. To test the feasibility, acceptance and effectiveness of this intervention

## Methods

**Figure 1:**
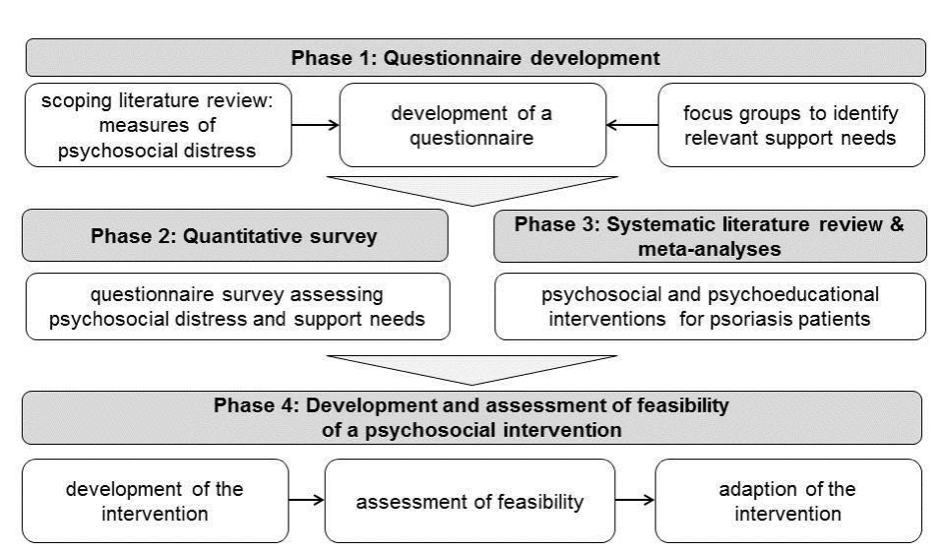
Flow-chart of study phases.

A multi-stage design with four phases combining quantitative and qualitative methodology will be conducted. It is displayed in Figure 1.

The project will be carried out at Department of Medical Psychology in cooperation with the Institute for Health Services Research in Dermatology and Nursing at the University Medical Center Hamburg-Eppendorf and the Psoriasis Center at the Department of Dermatology of the University Medical Center Schleswig-Holstein Campus Kiel in Germany.

### Phase 1: Questionnaire Development

A scoping review [35] will be conducted to identify measures to assess psychosocial distress in chronic conditions and skin diseases. Measures that focus on mental illnesses, like the Patient Health Questionnaire (PHQ-9) [36,37] to assess depression symptoms, the Generalized Anxiety Disorder 7 (GAD-7) [38] to measure anxiety symptoms or the Somatic Symptom Disorder (SSD-12) [39] to evaluate somatic symptoms will be used. Risk factors as alcohol consumption will be assessed, for example with the Alcohol Use Disorders Identification Test (AUDIT-C) [40]. Moreover, measures to evaluate the information needs and supportive care needs, as well as the utilization of supportive interventions, empowerment, and resources of psoriasis patients will be searched for and included. The study aims to complement the questionnaire with items, to detect patients that are in need for counseling and supportive interventions. Moreover, questions on sociodemographic data will be included.

Based on the results of the scoping review a complete questionnaire will be developed to assess the psychosocial burden of psoriasis patients. Following, two focus groups with n=10 patients with psoriasis in each focus group will be conducted to validate and complement the results of the scoping review.

#### Recruitment of the Patients

The patients will be consecutively recruited during consultations at the Psoriasis Centers of University Medical Center Schleswig-Holstein Campus Kiel and Institute for Health Services Research in Dermatology and Nursing of the University Medical Center Hamburg.

#### Inclusion Criteria

Adult patients (age ≥ 18) will be included. Patients need to be diagnosed with a mild, moderate, or severe psoriasis and need to agree by signing informed consent. Inclusion criteria will be checked from the treating physician, who will also fulfill a short questionnaire on the patients’ clinical data (severity of the psoriasis, presence of psoriasis arthritis or other comorbidity).

#### Focus Groups

One focus group will be conducted at the Psoriasis Center in Kiel and the second at the Department of Medical Psychology in Hamburg. Each focus group is planned to take 120 minutes and patients will receive 50€ for their participation. The main issue of the discussion will be to critically review the developed questionnaire in terms of feasibility and understanding with the patients and to add missing topics regarding information and supportive care needs.

#### Analysis

The focus groups will be recorded and transcribed. The results will show if any adaption of the questionnaire will be necessary or if further items should be developed and included based on the feedback of the patients. The final questionnaire will then be used in a quantitative survey in phase 2.

### Phase 2: Quantitative Survey

The questionnaire developed in phase 1 will be used in a cross-sectional survey to assess the extent of psychosocial distress and supportive care needs in patients with psoriasis.

#### Recruitment of Patients

Participants for phase 2 will be recruited again through consecutive consultations in the two cooperating dermatology centers.

**Figure 2:**
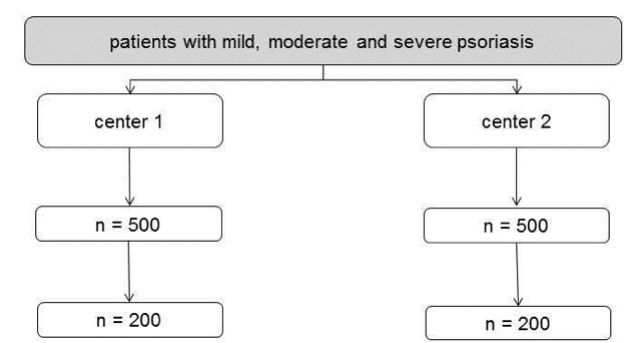
Survey flow-chart for the questionnaire. Calculated with an expected return rate of 40%.

#### Inclusion Criteria

The inclusion criteria are the same as for phase 1.

#### Sample Size

A sample size of N=400 is projected (Figure 2). Based on an expected return rate of 40% we will approach 1.000 patients, 500 per center.

#### Measures

Based on the results of the scoping review in phase 1 we expect to include measures on mental disorders, quality of life, psychosocial distress, health and risk behaviors, information needs and utilization of supportive interventions, needs for supportive care, empowerment, and resources of psoriasis patients. Moreover, sociodemographic data will be assessed. The health status in terms of psoriasis severity and comorbidity will be rated by the treating physician.

#### Analyses

The primary analyses for phase 2 will be descriptive (percentages, means, and standard deviations). The results of this phase will inform the focus of the treatment elements contained within the intervention.

### Phase 3: Systematic Literature Review and Meta-Analyses

To identify which psychosocial and psychoeducational interventions for patients with psoriasis already exist and which are effective, a systematic review will be conducted. The methods and results will be reported in accordance with the Preferred Reporting Items for Systematic Reviews and Meta-Analyses (PRISMA) statement [41]. First a search strategy will be defined followed by an electronic database search in the following databases: Cochrane Central Register of Controlled Trials (CENTRAL), MEDLINE, EMBASE, and PsycINFO. Two independent reviewers will stepwise screen titles, abstracts, and full-texts according to predefined inclusion criteria. Within the title and abstract screening all references that were rated as possibly relevant by one reviewer will be included. In the full-text screening disagreements will be resolved by discussion. In addition to the electronic search reference and citation tracking will be performed with all included references and systematic reviews that dealt with similar questions to check for further relevant references.

Data will be systematically extracted from the included references on the study population, details of the intervention and the outcomes. Furthermore, the risk of bias will be rated for all controlled trials by two independent reviewers according to the Cochrane Handbook for Systematic Reviews of Interventions [42]. Disagreement will be resolved by discussion as well.

For the most relevant patient-reported outcomes a meta-analysis will be performed on all controlled trials that assessed these outcomes. Primary outcome will be HrQoL. Additional outcomes will be chosen depending on whether there will be enough data for a meta-analysis. Furthermore, if the data allows it, subgroup analysis on HrQoL will be performed to compare different aspects of the interventions in their effectiveness.

#### Phase 4: Intervention Development and Assessment of Feasibility

Based on the results of the phases 2 and 3 a supportive intervention will be developed.

#### Intervention Development

Based on the needs assessment and the meta-analyses an intervention will be developed addressing the needs of the patients with the most promising intervention type, which can be face-to-face in a group or single setting or an internet-based approach. Internet-based interventions may reach larger parts of the patient population and allow a high degree of flexibility. Existing Internet-based programs (eg, for depression, alcohol consumption, or stress reduction) could be adapted for these patients or certain modules could be tailored [43,44]. Furthermore, barriers such as privacy concerns and fear of being stigmatized could be overcome by the anonymity of Internet-based interventions. Systematic reviews show favorable effects of Internet-based (cognitive behavioral) interventions for chronic somatic conditions [45,46], but research in dermatological conditions is rare [47]. However, a final decision about the type of intervention will be based on the patients care needs explored in phase 2 and the results of the meta-analysis in phase 3.

We plan to use qualitative methods to explore how professionals and people with psoriasis view the draft of the intervention. In the first step, a focus group with 6 health care professionals (four dermatologists and two psychologists) and 2 patient representatives (eg, of the “Deutscher Psoriasis Bund e.V.” [48] or self-help groups organized in Internet forums) are planned to discuss the developed intervention. The participants will be presented the intervention materials and asked to comment on content, acceptability, and usability. The experts will also be asked to discuss where in treatment procedure the intervention is most useful and realizable. The focus groups will be recorded and transcribed.

In the second step, n=8 patients with psoriasis will be sought using convenience sampling recruited from the participating clinics. Think aloud methodology [49] will be used, whereby participants will be asked to detail their ‘live’ reactions on the content and layout of the intervention. Patients will be encouraged to voice their views on all aspects of the intervention including likely usefulness of the content, appearance of the content, feasibility of following the advice etc. The intervention will be modified on the basis of these qualitative results.

#### Assessment of Feasibility

When the manual for the intervention is finally developed, the feasibility of the intervention will be assessed. The primary aim of this feasibility assessment will be to examine the potential of the intervention to cause clinical relevant changes on the patient’s side and to identify the methodological requirements for a future trial.

**Figure 3:**
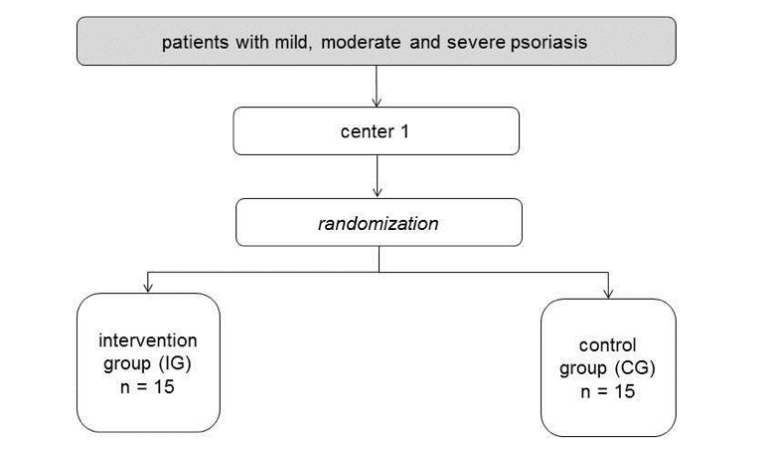
Survey flow-chart for the intervention.

Therefore, if it suits the intervention type, the study aims to recruit a sample of approximately 30 participants. These participants will be randomized into an intervention group and a control group (Figure 3). The measures for this assessment of effectiveness will be partly the same as used in phase two. Primary outcome will be patient reported distress and/or HrQoL. Additionally, information about the utility of the intervention and satisfaction with the intervention will be assessed. Open ended question will be included which ask about participants’ experience of using the intervention.

#### Analyses

The group means and standard deviations for the intervention group and control group before and after the intervention will be presented. In addition, inferential comparisons between both groups will be calculated. Yet due to the small population of the pilot study we do not expect to have enough power to reliably detect meaningful differences.

## Results

At the current state of the project the questionnaire development of phase 1 has been completed. The recruitment for the quantitative survey of phase 2 has been started and to date (19 of September 2017) n=185 patients have been included. The systematic review and meta-analysis of phase 3 are conducted simultaneously to phase 2 and results are expected to be completed soon. Phase 4 has not been started yet.

## Discussion

Previous research showed that patients with psoriasis are likely to suffer from psychosocial distress and various somatic and mental comorbidities [4]. Moreover, recent research found that intensity of pruritus experienced during psoriasis exacerbation can be related to stressful life events [29]. Therefore, screening for mental illnesses and needs for psychosocial information and care needs are clearly recommended within the treatment of psoriasis [4]. First studies were conducted to test psychosocial interventions for psoriasis; however, results on effectiveness were little to moderate and methodical flaws were criticized [32,33].

The described study will respond to the necessity of focusing on the psychosocial burden of this patient group in a multi-center project combining qualitative and quantitative methodologies. In four phases, relevant research questions will be addressed to close current research gaps. In the first phase an extensive questionnaire development will be conducted. This includes a scoping review on measures on psychosocial distress and information and supportive care needs as well as discussions on the questionnaire with patients in focus groups that will result in a comprehensive and validated questionnaire. In the second phase with the quantitative survey using the questionnaire developed in the prior phase data on the psychosocial distress and information and supportive care needs of patients from two psoriasis centers in Germany will be gained. Moreover, the pre-test of a screening measure will help to develop an instrument to screen for patients with support needs in future consultations.

In the third phase, a meta-analysis will be conducted that adds on prior systematic reviews by summarizing the effectiveness of previous studies on psychosocial/psychoeducative interventions for psoriasis patients. Up-to date, to our knowledge no systematic review including all psychosocial/psychoeducative interventions on psoriasis exists. Effects of these interventions have not been shown in a meta-analysis yet.

In the final phase, the results of the prior phases will be used for the development of psychosocial interventions that is tailored to the specific needs of this patient group. It is hoped that due to the comprehensive knowledge gained from the survey and the expert and patient focus groups the intervention will be found as effective.

The expected benefits from this study are to find out about the psychosocial support needs of patients with psoriasis and to add on previous research on patients’ goals in psoriasis treatment. Blome et al found that besides skin treatment related goals of psoriasis patients, like “to be healed of all skin defects,“ goals like “to be comfortable showing yourself more in public,” “to be able to have more contact with other people” or “to be more productive in everyday life,” are from high relevance for the patients [50]. The latter treatment goals are difficult to realize with somatic treatment only and again show the necessity of interdisciplinary treatment of this disease and the need for patient-centered care that values the individual patients needs [51] and, for example, in psychosocial offers for patients with psoriasis.

## Abbreviations

AUDIT-C: Alcohol Use Disorders Identification Test
CENTRAL: Cochrane Central Register of Controlled Trials
GAD-7: Generalized Anxiety Disorder 7
HrQoL: health-related quality of life
PHQ-9: Patient Health Questionnaire
PRISMA: Preferred Reporting Items for Systematic Reviews and Meta-Analyses
PsA: psoriatic arthritis
RCTs: randomized controlled trials
SSD-12: Somatic Symptom Disorder
